# Occurrence of False Positive Results for the Detection of Carbapenemases in Carbapenemase-Negative *Escherichia coli* and *Klebsiella pneumoniae* Isolates

**DOI:** 10.1371/journal.pone.0026356

**Published:** 2011-10-21

**Authors:** Peng Wang, Shudan Chen, Yan Guo, Zizhong Xiong, Fupin Hu, Demei Zhu, Yingyuan Zhang

**Affiliations:** 1 Institute of Antibiotics, Huashan Hospital, Fudan University, Shanghai, China; 2 State Key Laboratory of Genetic Engineering, Institute of Genetics, School of Life Sciences, Fudan University, Shanghai, China; 3 Department of Infectious Diseases, the First Affiliated Hospital, Anhui Medical University, Hefei, China; The University of Hong Kong, Hong Kong

## Abstract

Adequate detection of the production of carbapenemase in *Enterobacteriaceae* isolates is crucial for infection control measures and the appropriate choice of antimicrobial therapy. In this study, we investigated the frequency of false positive results for the detection of carbapenemases in carbapenemase-negative *Escherichia coli* and *Klebsiella pneumoniae* clinical isolates by the modified Hodge test (MHT). Three hundred and one *E. coli* and *K. pneumoniae* clinical isolates were investigated. All produced extended spectrum β-lactamases (ESBLs) but were susceptible to carbapenems. Antimicrobial susceptibility testing was performed by the disk diffusion and agar dilution methods. The MHT was performed using the standard inoculum of test organisms recommended by the CLSI. Genes that encoded ESBLs and carbapenemases were identified by PCR and DNA sequencing. Among the 301 clinical isolates, none of the isolates conformed to the criteria for carbapenemase screening recommended by the CLSI. The susceptibility rates for imipenem, meropenem, and ertapenem all were 100.0%, 100.0%, and 100.0%, respectively. Of the 301 *E. coli* and *K. pneumoniae* isolates, none produced carbapenemase. The MHT gave a positive result for 3.3% (10/301) of the isolates. False positive results can occur when the MHT is used to detect carbapenemase in ESBL-producing isolates and clinical laboratories must be aware of this fact.

## Introduction

The production of extended-spectrum β-lactamases (ESBLs) is the most common mechanism of resistance to third-generation cephalosporins in *Enterobacteriaceae* bacteria, especially *Escherichia coli* and *Klebsiella pneumoniae*. Carbapenems are the drugs of last resort to treat severe infections caused by pathogens that produce ESBLs [1]–[2]. Although the rates of resistance to carbapenems are low, they have been increasing [3]. During the past decade the spread of antibiotic resistance in *Enterobacteriaceae* isolates, especially the increase in *K. pneumoniae* carbapenemase (KPC)-producing isolates, has become a major concern worldwide [4]. Infections due to KPC-producing isolates have become an important challenge in healthcare settings [5]. The rapid emergence of carbapenem-resistant *Enterobacteriaceae* isolates in our hospital was revealed by bacterial surveillance data. The rate of resistance to imipenem in *K. pneumoniae* rose from 1.3% in 2006 to 11.1% in 2009, whereas the rate in *E. coli* rose from 0.3% 2006 to 1.0% in 2009 (data not shown).

The production of carbapenemases, especially KPC-type carbapenemases, is the most common mechanism for carbapenem resistance in *Enterobacteriaceae* isolates [6]–[7]. However, the detection of carbapenemases can be difficult because, for some carbapenemase-producing *Enterobacteriaceae*, the minimum inhibitory concentration (MIC) of carbapenem is high but still within the susceptible range as defined by the Clinical and Laboratory Standards Institute (CLSI) criteria [4], [8]–[9]. In 2009, the CLSI recommended the modified Hodge test (MHT) to screen for the production of carbapenemase in *Enterobacteriaceae* isolates with elevated MICs for carbapenems or reduced inhibition zones as measured by disc diffusion. The criteria were a diameter for the inhibition zones for meropenem and ertapenem of 19–21 mm and 16–21 mm, respectively, and MIC values of 2 µg/mL and 2–4 µg/mL, respectively (carbapenem breakpoints have been changed in M100-S20-U and M100-S21. According the new criteria, the initial screen test and the confirmatory test by MHT are no longer necessary for routine patient testing). Although the sensitivity and specificity of the MHT have been shown to exceed 90%, several studies have reported false positive or false negative results when this method was used to screen for carbapenemase in *Enterobacteriaceae* isolates [8], [10]. In this study, we investigated the rate of false positives obtained when the MHT was used to test ESBL-producing and carbapenemase-nagetive *E. coli* and *K. pneumoniae* clinical isolates that did not conform to the CLSI criteria for carbapenemase screening.

## Materials and Methods

### Bacterial strains

Three hundred and one non-duplicate clinical isolates were analysed. The isolates included 153 *E. coli* isolates and 148 *K. pneumoniae* isolates and had been collected from January to December 2008 in Huashan Hospital, Fudan University, Shanghai, China. All the isolates produced ESBLs, as confirmed by the CLSI phenotypic confirmatory test initially, but were susceptible to carbapenems (zones of inhibition of ≥19 mm in diameter for ertapenem and ≥16 mm for meropenem, respectively) (9). As control, 18 carbapenem-resistant, KPC-2 type carbapenemase-producing *K. pneumoniae* clinical isolates were used in this study (11).

### Antimicrobial susceptibility testing and the MHT

Antimicrobial susceptibility testing was performed using agar dilution methods recommended by the CLSI (9). Minimal inhibitory concentrations (MICs) of cefazolin, cefoxitin, cefotaxime, ceftriaxone, aztreonam, ceftazidime, cefepime, ertapenem, imipenem, meropenem, and piperacillin/tazobactam were determined in accordance with the CLSI criteria (9). The MHT was carried out on all isolates to detect carbapenemase using ertapenem and meropenem as described by the CLSI (9). A well characterized strain of *C. freundii* that produced IMP-9-type metallo-β-lactamase (MBL) and *E. coli* strain ATCC 25922 were used as positive and negative controls, respectively.

### Carbapenem inactivation assay

In order to determine whether resistance to imipenem and meropenem was likely caused by production of carbapenemase, a disk diffusion bioassay was performed on Mueller-Hinton agar with imipenem, meropenem and ertapenem disks as described by Yigit et al [12].

### Genotypic detection of β-Lactamase

The β-lactamase genes *TEM*, *SHV*, *PER*, *SFO*, *VEB*, *CTX-M* and *GES*, and carbapenemases including *KPC*, *VIM*, *IPM*, *SPM*, *SME*, *GIM*, *NMC*, *IMI*, *IND* and *OXA*, were investigated in all of clinical strains using previously described primers [13]–[15]. Conserved primers, forward primer KPC-F (5′-AGGACTTTGGCGGCTCCAT-3′) and reverse primer KPC-R (5′-TCCCTCGAGCGCGAGTCTA-3′), were designed against the *KPC* gene to detect the *KPC*-1 to *KPC*-11 sequences according to the published nucleotide sequences in Genbank, which encode different KPC-type carbapenemases and produced a 748-bp PCR product. PCR amplicons were sequenced, and the DNA sequences obtained were compared to the available sequences via National Center for Biotechnology Information BLAST search.

### Analysis of outer membrane proteins

The outer membrane proteins of all isolates with false-positive result on MHT were examined by sodium dodecyl sulfate polyacrylamide gel electrophoresis, as described previously [14].

### Ethics Statement

As this study was focused on bacterial isolates collected from routine samples, approval from ethics committee at Fudan University was not necessary. Neither was there any need for informed consent procedures as no extra sampling was performed and no personal data was stored in relation to the isolates.

## Results

### Antimicrobial susceptibility testing

The results of the antimicrobial susceptibility testing indicated that, for the 301 isolates tested, the inhibition zones for meropenem and ertapenem all had a diameter greater than 22 mm, and the MIC values were all below 1 µg/mL. Therefore, none of the isolates conformed to the CLSI criteria for carbapenemase screening (namely, diameters for meropenem and ertapenem of 19–21 mm and 16–21 mm, respectively, and MIC values of 2 µg/mL and 2–4 µg/mL, respectively.). None of the isolates was found to be susceptible to cefazolin and 99.7%, 62.1%, 48.2%, and 19.9% were resistant to cefotaxime, ceftazidime, cefepime, and cefoxitin, respectively. The susceptibility rates for imipenem, meropenem, and ertapenem were 100.0%, 100.0%, and 100.0%, respectively (Table 1).

**Table 1:** Antimicrobial activities of various antimicrobial agents against 301 *E. coli* and *K. pneumoniae* isolates.

Drug	MIC (µg/ml)			S (%)	R (%)
	Range	MIC_50_	MIC_90_		
Imipenem	≤0.06–0.25	≤0.06	≤0.06	100.0	0.0
Meropenem	≤0.06–0.25	≤0.06	≤0.06	100.0	0.0
Ertapenem	≤0.06–0.25	≤0.06	0.125	100.0	0.0
Cefepime	0.125–>128	16	64	30.9	48.2
Ceftazidime	0.25–>128	32	>128	27.9	62.1
Cefotaxime	0.125–>128	128	>128	0.3	99.7
Ceftriaxone	0.125–>128	>128	>128	0.3	99.7
Cefoxitin	1–>128	8	128	62.8	19.9
Cefazolin	128–>128	>128	>128	0.0	100.0
Piperacillin-tazobactam	0.5–>128	8	>128	65.8	23.9
Aztreonam	0.25–>128	64	>128	8.6	83.1

### PCR amplification of β-lactamase genes and the MHT

**Figure 1:**
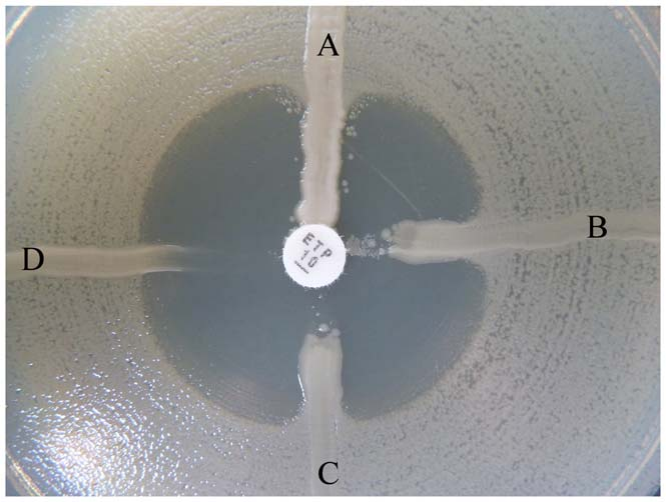
False-positive result on MHT. A, K2821 *K. pneumoniae* (KPC-2 carbapenemase producer). B, 08-438 *E.coli*. C, 08-97 *K. pneumoniae*. D, *E. coli* ATCC 25922.

Of the 301 *E. coli* and *K. pneumoniae* isolates, none produced carbapenemase. Genes that encoded the CTX-M-type ESBLs were detected in 280 (93.0%) of the isolates, among which CTX-M-14, CTX-M-15, CTX-M-25, CTX-M-14 coupled with CTX-M-15, and CTX-M-14 coupled with CTX-M-25 type ESBL were found in 135 (48.2%), 112 (40.0%), 2 (0.7%), 28 (10.0%), and 3 (1.1%) of the isolates, respectively. Among 301 isolates, 18.3% were producing more than two types of ESBLs, such as TEM-type and CTX-M-type ESBLs, SHV-type and CTX-M-type ESBLs (Table 2). Although all the isolates were sensitive to carbapenems and carbapenemase-negative, the MHT yielded a positive result (Figure 1) for 3.3% (10/301) of the isolates and all the MHT-positive isolates (except one isolate was SHV-12 type ESBL producer) were CTX-M type ESBL producing (Table 3). Among 18 carbapenem-resistant *K. pneumoniae* clinical isolates, all were produced KPC-2 type carbapenemase and all were producing a positive result of MHT.

**Table 2:** Distribution of ESBL genes among the 301 *E. coli* and *K. pneumoniae* isolates.

ESBL genes	Genotype	Number	%
SVH-type ESBLs	SHV-2, SHV-12, SHV-28, SHV-31	8	2.7
CTX-M-type ESBLs	CTX-M-14, CTX-M-15, CTX-M-25	243*	80.7
TEM + CTX-type ESBLs	TEM-40, TEM-135, CTX-M-14, CTX-M-15	3	1.0
SHV + CTX-M-type ESBLs	SHV-2, SHV-12, SHV-28, SHV-31, CTX-M-14, CTX-M-15, CTX-M-25	34	11.3
Negative**		13	4.3
Total		301	100.0

*: 9.0% (22/243) isolates were producing both CTX-M-14 and CTX-M-15 type ESBL, and 1.2% (3/243) isolates were producing both CTX-M-14 and CTX-M-25 type ESBL.

**: We did not detected the TEM, SHV or CTX-M type ESBLs among these isolates.

**Table 3:** The characteristics of the 10 false-positive isolates by MHT.

Number	Organism	MIC (mg/L)				ESBLs genes
		Cefotaxime	Ceftazidime	Cefoxitin	Ertapenem	
08-309	*E. coli*	128	2	4	≤0.06	CTX-M-14
08-318	*E. coli*	>128	128	8	≤0.06	CTX-M-15
08-438	*E. coli*	128	32	>128	≤0.06	CTX-M-15
08-547	*E. coli*	64	4	16	≤0.06	CTX-M-14
08-1002	*E. coli*	128	16	8	≤0.06	CTX-M-15
08-1039	*E. coli*	128	2	4	≤0.06	CTX-M-14
08-97	*K. pneumoniae*	>128	>128	4	≤0.06	CTX-M-15, SHV-28
08-1105	*K. pneumoniae*	>128	128	4	≤0.06	CTX-M-15
08-466	*K. pneumoniae*	64	>128	128	0.125	SHV-12
08-893	*K. pneumoniae*	>128	128	4	≤0.06	CTX-M-15, SHV-61

### Carbapenem inactivation assay

A carbapenem inactivation assay performed on all 301 carbapenems-susceptible isolates indicated that alterations in shape of the zones of inhibition around the test organism were not observed, suggesting no carbapenemase was involved in hydrolysis of carbapenems in these isolates. However The assay of all 18 KPC-2 type carbapenemase-producing *K. pneumoniae* isolates were positive.

### Analysis of outer membrane proteins

**Figure 2:**
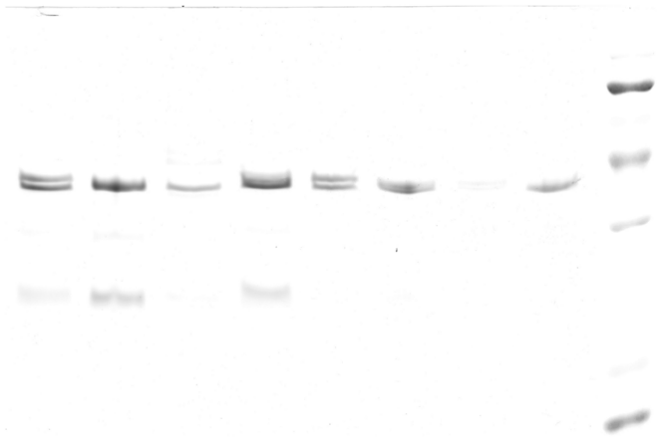
1. *E.coli* ATCC 25922, 2. 08-309 *E.coli*, 3. 08-318 *E.coli*, 4. 08-438 *E.coli*, 5. *E.coli* ATCC 25922, 6. 08-547 *E.coli*, 7. 08-1002 *E.coli*, 8. 08-1039 *E.coli*, M. Marker.

**Figure 3:**
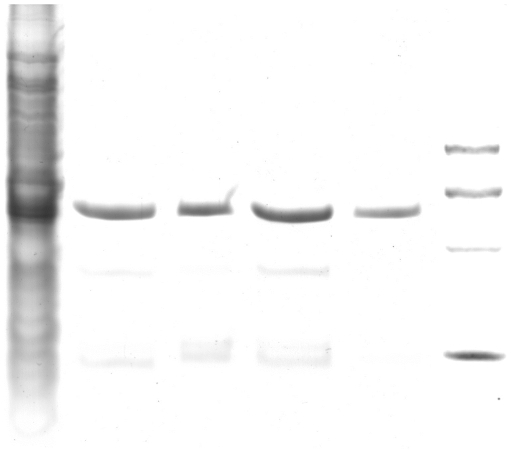
1. K1933 *K. Pneumoniae* (Sensitive to cefazolin, no ESBL and carbapenemase producer), 2. 08-97 *K. Pneumoniae*, 3. 08-1105 *K. Pneumoniae*, 4. 08-466 *K. Pneumoniae*, 5. 08-893 *K. Pneumoniae*, M. Marker.

Analysis of the outer membrane porin proteins by sodium dodecyl sulfate polyacrylamide gel electrophoresis showed that all 10 isolates (except 08-438 *E.coli*) exhibited loss of one of porin protein compared with the sensitive strains (Figure 2 and Figure 3). This suggests that the outer membrane porin proteins might play an important role in resulting to the false-positive result.

## Discussion

Given the increasing prevalence of carbapenemase-producing *Enterobacteriaceae* isolates worldwide [4], simple and accurate tests are needed to detect isolates that produce carbapenemase. For this purpose, the CLSI have recommended the MHT for the detection of carbapenemase in *Enterobacteriaceae* isolates. Although the MHT is a simple method for detecting carbapenemase-positive isolates, false positive results are obtained occasionally [8], [10]. In June 2010, CLSI changed the carbapenem breakpoints and reviewed the recommendation on carbapenemase detection by MHT among *Enterobacteriacea* (M100-S20-U) and informed that MHT would not be recommended, except for epidemiological or infection control purposes.. In this study, we used carbapenem-susceptible *E. coli* and *K. pneumoniae* isolates that did not conform to the CLSI criteria for screening for the presence of carbapenemase. False positive results for the MHT were obtained among 3.3% of the isolates. We presume that the false positive results are probably due to low-level hydrolysis of ertapenem by ESBLs, particularly those of the CTX-M type, because we found that 97.3% of the isolates in our study produced CTX-M-type ESBLs [10], [16].

Although the false positive results were observed in our study, MHT is not recommended (and never was) to detect carbapenemase among isolates that yielded negative-result on screening test. Whether for epidemiological or infection control purposes, adequate detection of the production of carbapenemase in *Enterobacteriaceae* isolates is crucial for infection control measures and the appropriate choice of antimicrobial therapy [8]. To improve the detection of carbapenemase-producing *Enterobacteriaceae* in clinical microbiology laboratories, several phenotypic tests to detect KPCs have been developed [17]–[20]. In addition to the MHT, a second phenotypic method has been shown to be promising for the identification of KPCs. This method utilizes boronic acid and 3-aminophenyl boronic acid-based compounds, and has proved to be highly sensitive and specific for the detection of KPCs.

In summary, as reported, CTX-M-type ESBLs might be hydrolysing the ertapenem used in the assay [16]. Clinical laboratories must be aware that false positive results may occur, especially in geographical areas where the incidence of isolates that produce CTX-M-type ESBLs is relatively high, such as the Asia-Pacific region [21]. Hence, further work is necessary to determine the frequency of false positive results when the MHT is used to detect carbapenemase production in *Enterobacteriaceae* isolates and more accurate methods should be developed for use in clinical microbiology laboratories.
